# Safety engineered injection devices for intramuscular, subcutaneous and intradermal injections in healthcare delivery settings: a systematic review and meta-analysis

**DOI:** 10.1186/s12912-015-0119-1

**Published:** 2015-12-30

**Authors:** Alain C. Harb, Rami Tarabay, Batoul Diab, Rami A. Ballout, Selma Khamassi, Elie A. Akl

**Affiliations:** Department of Anaesthesiology, American University of Beirut, Beirut, Lebanon; Lebanese University, Beirut, Lebanon; American University of Beirut, Beirut, Lebanon; World Health Organization, Geneva, Switzerland; Department of Internal Medicine, American University of Beirut, Beirut, Lebanon

**Keywords:** Systematic review, Meta-analysis, Health care worker, Needlestick injuries, Sharps injury prevention syringes, Reuse prevention syringes, HIV, HBV, HCV

## Abstract

**Background:**

Occupational sharps injuries are associated with transmission of bloodborne viruses to healthcare workers, including hepatitis B virus (HBV), hepatitis C virus (HCV), and human immunodeficiency virus (HIV). Similarly reuse of syringes in healthcare settings might transmit these infections between patients. The objective of this study was to systematically review the evidence about the effects of the use by health care workers of two types of safety engineered injection devices, when delivering intramuscular, subcutaneous, or intradermal injectable medications: sharps injury protection syringes and reuse prevention syringes.

**Methods:**

We included both randomized and non-randomized studies comparing safety syringes to syringes without safety features. Outcomes of interest included needlestick injuries, and HIV, HBV and HCV infections amongst HCWs (for sharps injury prevention syringes) and patients (for reuse prevention syringes). When possible, we conducted meta-analyses using a random-effects model. We tested results for heterogeneity across studies using the I statistic. We assessed the quality of evidence by outcome using the GRADE methodology.

**Results:**

We included nine eligible studies: six assessed devices that qualify as sharps injury prevention devices, and three assessed devices that qualify as both injury prevention devices and reuse prevention devices. Eight studies were observational while one was randomized. All studies assessed a single outcome: needle stick injuries among healthcare workers. For sharp injury prevention syringes, the meta-analysis of five studies resulted in a pooled relative risk of 0.54 [0.41, 0.71] for the effect on needlestick injuries per healthcare worker. The associated quality of evidence was rated as moderate. For reuse prevention syringes, data from one study provided a relative risk of 0.40 [0.27, 0.59] for the effect on needlestick injuries per healthcare worker. The associated quality of evidence was rated as moderate. We identified no studies reporting on the effect on the reuse of syringes.

**Conclusions:**

We identified moderate quality evidence that syringes with sharps injury prevention feature reduce the incidence of needlestick injuries per healthcare worker. We identified no studies reporting data for the remaining outcomes of interest for HCWs. Similarly we identified no studies reporting on the effect of syringes with a reuse prevention feature on the reuse of syringes or on the other outcomes of interest for patients.

**Electronic supplementary material:**

The online version of this article (doi:10.1186/s12912-015-0119-1) contains supplementary material, which is available to authorized users.

## Background

Healthcare workers (HCWs) exposure to bloodborne pathogens from sharps injuries, primarily needlesticks, is a serious occupational problem. The World Health Organization (WHO) reported that more than three million HCWs were exposed to bloodborne pathogens from percutaneous exposure in the year 2000 across the world [1]. In the United States alone, and according to the Centers for Disease Control and Prevention (CDC), hospital-based HCWs suffer about 385,000 such injuries annually. This amounts to an average of 1000 injuries per day [2]. In the United Kingdom, sharps injuries account for 17 % of accidents to the National Health Services staff [3].

Occupational sharps injuries are associated with transmission of bloodborne viruses, the most serious and potentially fatal of which are hepatitis B virus (HBV), hepatitis C virus (HCV), and human immunodeficiency virus (HIV) [4, 5]. They are also associated in the transmission of more than 20 other pathogens [4, 5]. It has been estimated that occupational sharps injuries are responsible for 32 % of HBV infections, 40 % of HCV infections, and 5 % of HIV infections [6]. The HCWs risk of sharp injury related infection is relatively high in Africa, where HIV is prevalent and HBV is endemic amongst the patient population [7].

The burden of sharp injuries affects both HCWs and healthcare institutions. Sharp injury related blood-borne infections lead to absenteeism, morbidity and, mortality among HCWs [8]. They may also induce psychological stress, and negatively affect the personal and work life of HCWs [9, 10]. Hospitals also suffer from costs related to testing, treatment, and lost working time [11].

Reuse of syringes in healthcare settings can transmit these infections between patients. In the year 2000, the reuse of injection equipment accounted for 32, 40 and 5 % of new HBV, HCV, and HIV infections worldwide [12]. The estimated burden related to this practice is around 9.18 million disability-adjusted life years (DALYs) between the years 2000 and 2030 [13].

One of the suggested interventions to reduce sharps related injuries is the use of safety-engineered devices, which have mechanisms to prevent percutaneous injuries [14]. Indeed, introducing the use of these devices may prevent sharp injuries and the associated bloodborne infections [15]. Safety features of safety-engineered devices are designed to shield the needle or non-needle sharp object after use. There are also two main types of safety syringes:

1. Sharps injury prevention syringes: these use different mechanisms e.g. self-retractable needles, internal blunt needles, or external shielding
2. Reuse prevention– syringes: these include a reuse prevention feature e.g. metal clip to block the plunger once the injection is given, the plunger breaks etc. (making them unusable after initial use).

We conducted this study in preparation for the development of WHO policy guidance on use of safety-engineered devices by healthcare workers to deliver IM, SC and ID injections. The objective was to systematically review the evidence about the effects of the use by health care workers of two types of safety devices: sharps injury prevention syringes and reuse prevention syringes.

The specific review questions were:

1. What are the benefits and harms of sharps injury prevention syringes versus single use disposable syringes when used by healthcare workers to deliver intramuscular, subcutaneous or intradermal injections to patients?
2. What are the benefits and harms of reuse prevention syringes versus single use disposable syringes when used by healthcare workers to deliver intramuscular, subcutaneous or intradermal injections to patients?

## Methods

The study consisted of a review of the literature and did not involve any ‘human subjects’.

### Protocol and registration

We developed two separate protocols for sharp injury prevention syringes and reuse prevention syringes. We registered the protocols with the International database of prospectively registered systematic reviews in health and social care (PROSPERO) [10, 11].

### Eligibility criteria

#### Types of studies included

We included both randomized trials and non-randomized studies including:

- Cohort studies
- Case control studies
- Before and after studies
- Time-series analysis

We excluded scientific meeting abstracts, research letters, qualitative studies, letters to the editor, reviews, case reports, and case series.

#### Types of participants and settings

We included studies of healthcare workers delivering intramuscular, subcutaneous, or intradermal injectable medications. We were not interested in non-healthcare settings (e.g., illicit drug use, patients using insulin pen needles). We were not interested in other types of injections (e.g., phlebotomy or intravenous, articular, intra cardiac, and intra peritoneal injections).

#### Types of interventions

We included studies assessing the introduction of a safety device (sharp injury prevention syringes or reuse prevention syringes) into a healthcare setting. This introduction could have been accompanied by training of HCWs. Eligible sharp injury prevention syringes included: retractable syringes; needle shields, and recapping devices; needleless injectors; needle-safety devices; Eligible reuse prevention syringes included: auto-disable syringes (earlier called “auto-destruct syringes”) (ISO 7886–3), typically meant for vaccination; reuse prevention devices for therapeutic injections (ISO 7886–4); and pre-filled syringe with reuse prevention feature.

Ineligible devices included: intravenous devices; needless adaptors; fistula needle; IV catheters; winged steel needle; implantable port needles; suture needles; all blood collection devices (lancet devices, vacuum tubes for blood collection devices, an arterial blood syringes).

We included studies assessing the introduction of both eligible and ineligible devices as long as they reported data for eligible devices separately. We included the studies not reporting data for eligible devices separately in a sensitivity analysis.

#### Types of comparison(s)

We included studies comparing one of the interventions of interest to using a device without a safety feature, such as the ‘single use disposable syringes’ (ISO 7886–1).

#### Outcomes

We included studies assessing at least one of the following outcomes for sharps injury prevention syringes:

- HIV, HBV, and HCV infections amongst HCWs
- Other blood-borne infections (e.g. viral hemorrhagic fevers) amongst HCWs
- Abscesses (septic, aseptic) amongst HCWs
- Needlestick injuries amongst HCWs
- Quality of life amongst HCWs
- Social impact (e.g., stigma, job loss) amongst HCWs

We included studies assessing at least one of the following outcomes for reuse prevention syringes:

- Reuse of syringes
- HIV, HBV, and HCV infections amongst patients.
- Other blood-borne infections (e.g. viral hemorrhagic fevers) amongst patients
- Quality of life amongst patients
- Social impact (e.g., stigma, loss of job) amongst patients
- Needlestick injuries, HIV, HBV, and HCV infections amongst HCWs

Any positive impact on those outcomes would be considered as a benefit, while any negative impact on these same outcomes would be considered as harm.

### Literature search

We used the OVID interface to electronically search in October 2013 the following databases, starting with the dates of their inception: MEDLINE, EMBASE, and CINAHL. OVID is a platform that provides access to online bibliographic databases, academic journals, and other products, chiefly in the area of health sciences. We also electronically searched in October 2013 the Cochrane Central Register of Controlled Trials (CENTRAL). We did not use any study design filter, as we wanted to capture different types of study designs, particularly both randomized and non-randomized studies. We did not use language or date restrictions. Additional file 1 lists the search strategies used. We removed duplicates using the ‘find duplicates’ function in the EndNote software. In addition to the search of electronic databases, we reviewed the references lists of relevant papers; contacted experts; and searched personal files for both published and unpublished studies.

### Selection process

The reviewers were organized into two teams of two. Prior to starting the selection process, we conducted calibration exercises to clarify the eligibility criteria. We reviewed 100 citations with every exercise. We achieved agreement by the third exercise. At that point, the two review teams started screening titles and abstracts of identified citations in duplicate and independently. We obtained the full texts for citations judged as potentially eligible by at least one reviewer. Then, the two review teams screened the full texts in duplicate and independently for eligibility. They used a standardized and pilot tested full text screening form. The reviewers compared results and resolved disagreements by discussion or with the help of a third reviewer. We calculated agreement between reviewers for full text screening using the kappa statistic.

### Data abstraction process

The two review teams abstracted data from eligible studies in duplicate and independently. They used a standardized and pilot tested data abstraction form with detailed instructions. Then, the reviewers compared results and resolved disagreements by discussion or with the help of a third reviewer. The data items abstracted included:

- Description of the study device
- Study design
- Characteristics of participants and setting
- Description of the intervention
- Description of the control
- Outcomes assessed and statistical results
- Funding and disclosed conflicts of interest

### Risk of bias assessment

The two review teams assessed the risk of bias in each study in duplicate and independently. They used a standardized and pilot tested data abstraction form with detailed instructions. Then the reviewers compared results and resolved disagreements by discussion or with the help of a third reviewer. According to recommendations outlined in the Cochrane Handbook, we used the following criteria for assessing the risk of bias in randomized studies:

- Inadequate sequence generation;
- Inadequate allocation concealment;
- Lack of blinding of participants, providers, data collectors, outcome adjudicators, and data analysts
- Incompleteness of outcome data;
- Selective outcome reporting, and other bias.

We used the following criteria for assessing the risk of bias in non-randomized studies:

- Failure to develop and apply appropriate eligibility criteria
- Flawed measurement of exposure
- Flawed measurement of outcome
- Failure to adequately control confounding
- Incomplete follow-up

We judged each potential source of bias as high, low or unclear risk of bias.

### Data synthesis

For categorical data, we calculated the relative risk (RR) for each outcome for each study. RR refers to the risk in the intervention group or period (e.g., the introduction of a safety device) relative to the risk in the control group or period (e.g., using a device without a safety feature).

We assumed that variability in the population, interventions, control, and outcome measurements across studies will introduce heterogeneity in findings across those studies. To minimize this heterogeneity, we analyzed separately data for sharps injury prevention syringes and data for reuse prevention syringes. Also we analyzed separately different measurement of the same outcome, e.g., NSI per device and NSI per HCW. In order to deal with residual heterogeneity, we then pooled the results of studies using Mantel-Haenszel method (with hybrid inverse variance weighting) to accommodate random-effects. We did not choose the fixed-effects model because it assumes a common effect size, and it is inaccurate with a very small number of studies [16, 17]. In a random-effects meta-analysis the treatment effects for the individual studies are assumed to vary around some overall average treatment effect.

We tested results for heterogeneity across studies using the I statistic. We considered heterogeneity to be substantial if I is greater than 50 %. We planned to create inverted funnel plots of individual study results plotted against sample size in order to check for possible publication bias.

### Sensitivity analysis

We identified two studies that assessed both devices for intravenous injections or phlebotomy and devices for intramuscular, subcutaneous or intradermal injections, without providing data separately for the different types of devices [18, 19]. In a post hoc decision, we included these studies in the main analysis but excluded them in a sensitivity analysis to test the impact of their data on the final results. We used the freely available software RevMan 5.1.0 for all analyses [20].

### Subgroup analysis

We planned to explain heterogeneity, if present, by conducting subgroup analyses based on the following factors: route of injection (intramuscular, intradermal, subcutaneous), the type of device, level of expertise of HCWs, and time of injury (before, during, or after the injection). In order to assess the effects of reuse prevention devices, and given we did not identify any study assessing a device that is purely a reuse prevention device, we conducted a subgroup analyses of studies of devices that qualified as both reuse prevention devices and sharps injury prevention devices [15, 21, 22].

### Quality of evidence assessment

We assessed the quality of evidence by outcome using the GRADE methodology [23].

We produced a GRADE Evidence Profile to summarize the statistical findings and quality of evidence by outcome.

## Results

### Study selection

**Fig. 1:**
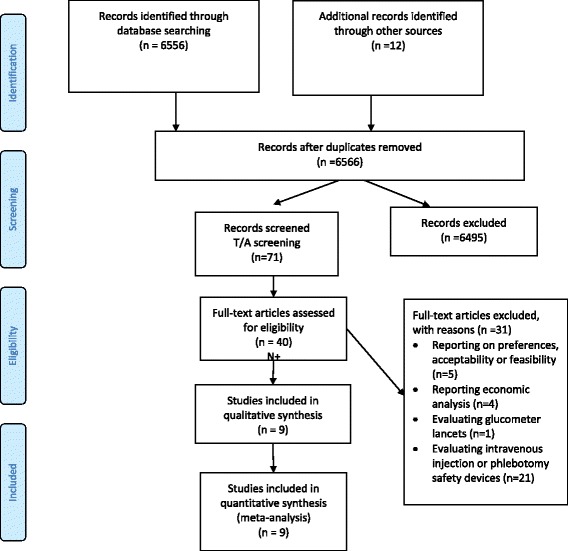
Study flow

Figure 1 shows the study flow. Out of a total of 6566 identified citations, we judged nine as eligible for this systematic review [15, 18, 19, 21, 22, 24–27]. Agreement between reviewers for full text screening was high (kappa statistic = 1). Additional file 2 provides the list of the 32 excluded studies with the following reasons for exclusion: reporting on preferences, acceptability or feasibility (*n* = 5); reporting economic analysis (*n* = 4); evaluating glucometer lancets (*n* = 1); reporting data not in healthcare setting (*n* = 1); and evaluating intravenous injection or phlebotomy safety devices (*n* = 22) [10, 28–57].

### Study characteristics

Additional file 3 provides the list of the nine included studies with detailed description of their characteristics.

#### Type of injection

Out of the nine included studies, five assessed devices for intravenous injection or phlebotomy, in addition to intramuscular, subcutaneous or intradermal injection devices [15, 18, 19, 24, 27]. Of these five studies, three provided data separately for the different types of devices [15, 24, 27]. The remaining two studies reported data combined for the different type of devices [18, 19].

#### Types of devices

Out of these nine studies, six assessed devices that qualify as sharps injury prevention devices [18, 19, 24–27], while three assessed devices that qualify as both sharps injury prevention devices and reuse prevention devices [15, 21, 22].

No studies included a comparison between active and passive devices. Two studies reported SIDs with active safety features [22, 26], two studies reported SIDs with passive safety features [19, 21] and 5 studies had SIDs with both active and passive or unspecified safety features [15, 18, 24, 25, 58].

#### Device brand

Six specified the device brand and/or the manufacturer:

- Monoject™ Safety Syringe by Sherwood Medical [21]
- VanishPoint® by Retractable Technologies, Inc. [15, 22]
- SafetyGlide™ devices [19], Eclipse™ [25, 27] by Becton Dickinson
- Surshield™ device by Terumo [27]

The remaining three studies specified neither the brand nor the manufacturer [18, 24, 26].

#### Funding

Five studies reported their funding sources as follows:

- Sherwood Medical; [21]
- Becton Dickinson; [19]
- National Institute of Allergy and Infectious Diseases; the Centers for Disease Control and Prevention; and the Prevention Epicenters; [24]
- Directorate General of Public Health of the Autonomous Community of Valencia, Spain; [27]
- Dutch Ministry of Social Affairs and Employment support; [25]

The remaining four studies did not report their funding sources [15, 18, 22, 26]. Two of these studies evaluated VanishPoint® by Retractable Technologies, Inc. [15, 22] while the other two specified neither the brand nor the manufacturer of the device under evaluation [18, 26].

#### Conflicts of interest

Two studies reported that their authors had no conflicts of interest [26, 27] The remaining studies did not provide conflicts of interest disclosures.

#### Study design

One study was a cluster prospective randomized controlled trial [25]. The remaining eight studies were non-randomized and used a before and after study design. Out of these 8 studies, five collected data prospectively [15, 19, 21, 24, 27] while three collected the data retrospectively [18, 22, 26].

#### Settings

Included studies were all conducted in the following high income countries: Australia (*n* = 1); [15] Germany (*n* = 1); [26] Netherlands (*n* = 1); [25] Spain (*n* = 1); [27] United Kingdom (*n* = 1); [19] and United States (*n* = 4) [18, 21, 22, 24].

#### Intervention

Interventions consisted of the introduction of the safety devices detailed above under “Device brand”. For seven studies, it was reported that healthcare workers received some form of educational intervention with regards to using the safety devices [19, 21, 22, 24–27].

#### Control

All included studies reported using “standard”, “conventional” or “traditional” syringes in the ‘before’ phase. One study reported conducting a needle safety workshop in the control group [25].

#### Outcomes

All studies assessed needle stick injuries among healthcare workers. None of the studies reported valuable data on any of the other outcomes of interest. Whitby et al. reported the following: “No significant increase in bloodstream infections was detected during the study period” [15].

### Risk of bias within studies

**Fig. 2:**
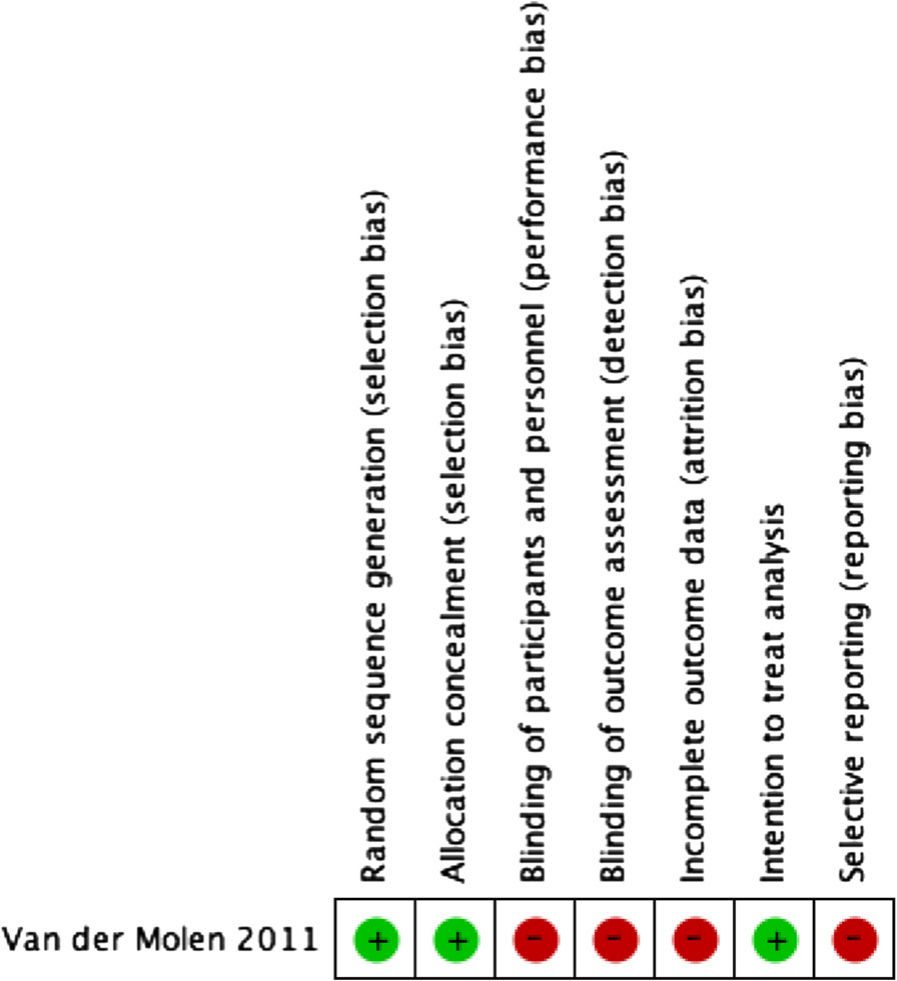
Risk of bias summary: review authors’ judgements about each risk of bias item for the included randomized study

Additional file 4 details and Fig. 2 summarizes the risk of bias in the included randomized study. The trial was at high risk of bias in relation to four out of 7 criteria assessed.

**Fig. 3:**
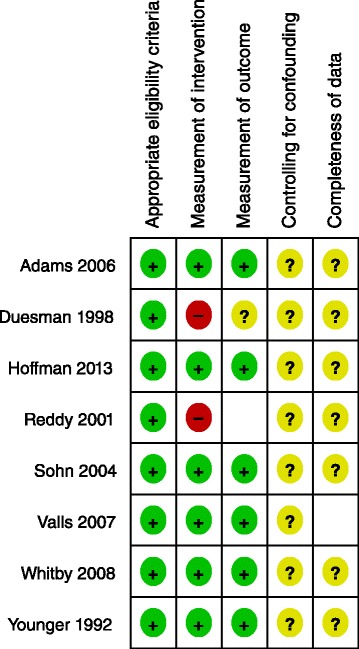
Risk of bias summary: review authors’ judgements about each risk of bias item for each included non-randomized study

**Fig. 4:**
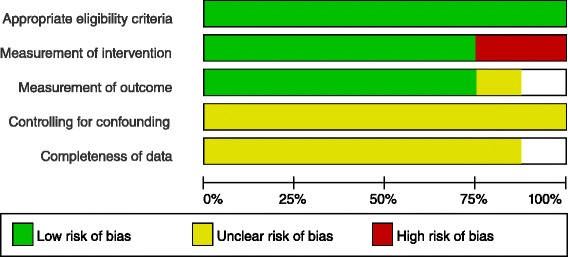
Risk of bias graph: review authors’ judgements about each risk of bias item presented as percentages across all included non-randomized studies

Additional file 5 details and Figs. 3 and 4 summarize the risk of bias in the included non-randomized studies. While the non-randomized studies were generally at low risk for bias in relation to the appropriateness of eligibility criteria, measurement of the intervention, and measurement of the outcome, they were all at unclear risk of bias in relation to dealing with confounding and completeness of data.

### Meta-analyses for sharps injury prevention syringes

Eligible studies reporting on the needlestick injuries (NSI) used two main types of statistics: incidence of NSI per device used (or purchased) [19, 21, 22, 27], and incidence of NSI per healthcare worker [15, 18, 24–26]. We conducted separate meta-analyses for these different statistics. One study reported incidence of NSI per patient [27].

NSIs reported for all studies were converted to a per year basis.

### Needlestick injuries

#### NSI per device

**Fig. 5:**
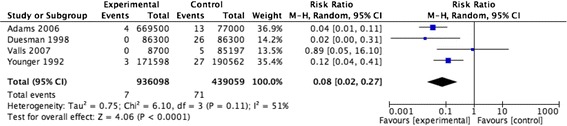
NSI per device for injury prevention devices

**Fig. 6:**
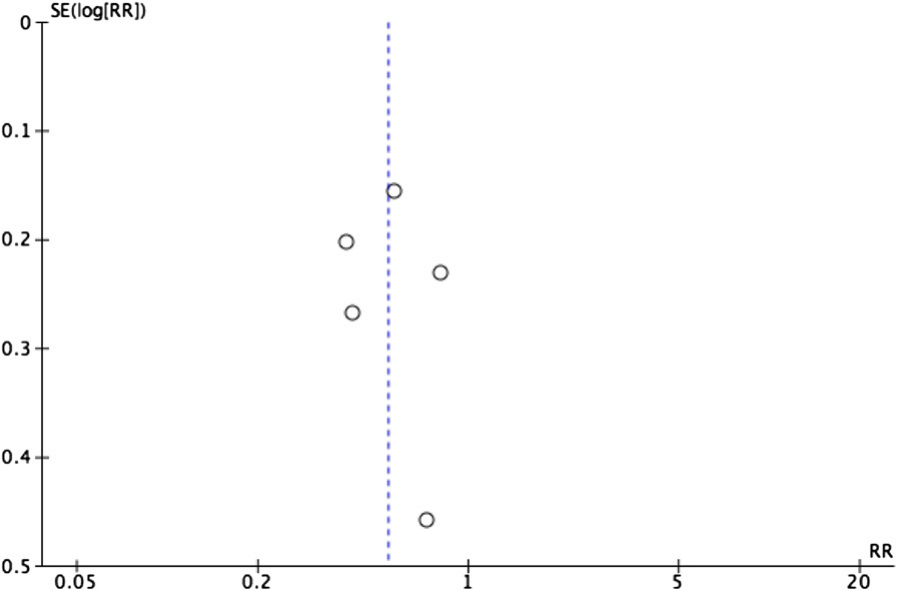
Inverted funnel plot for the outcome: NSI by device for injury prevention devices

The meta-analysis of four studies resulted in a pooled relative risk of 0.08 [95 % Confidence Interval (CI) 0.02, 0.27] (Fig. 5) [19, 21, 22, 27]. The I value was 51 %. The inverted funnel plot, although based on only five studies, did not suggest any publication bias (Fig. 6). The sensitivity analysis excluding the one study that did not report separately data for devices for intramuscular, subcutaneous or intradermal injection devices, [19] resulted in a pooled relative risk of 0.12 [95 % CI 0.02, 0.75] and I value of 50 %.

#### NSI per healthcare worker

**Fig. 7:**
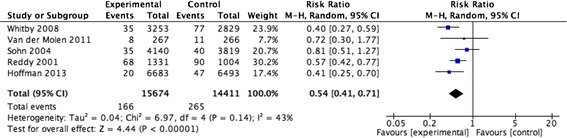
NSI per healthcare worker for injury prevention devices

The meta-analysis of five studies resulted in a pooled relative risk of 0.54 [0.41, 0.71] (Fig. 7) [15, 18, 24–26]. The I value was 43 %. The quality of evidence was rated as moderate (Table 1). Of note, one of the included studies reported data based on a time-series analysis, but we opted to analyze it as a before and after study in order to include it in the meta-analysis. The sensitivity analysis excluding the one study that did not report separately data for devices for intramuscular, subcutaneous or intradermal injection devices [18], resulted in a pooled relative risk of 0.53 [0.36, 0.79] and I value of 56 %. Restricting the analysis to the only included randomized trial resulted in a relative risk of 0.72 [0.30, 1.77].

**Table 1:** Evidence profile for sharp injury prevention syringes versus single use disposable syringes when used by healthcare workers to deliver intramuscular, subcutaneous or intradermal injections

Quality assessment							№ of patients		Effect		Quality	Importance
№ of studies	Study design	Risk of bias	Inconsistency	Indirectness	Imprecision	Other considerations	IM/SC/I Injury prevention safety devices	No safety devices	Relative (95 % CI)	Absolute (95 % CI)		
HIV, HBV and HCV infections amongst HCWs: not reported												
												Critical
NSI per HCW												
6	Observational	Not serious	Not serious	Not serious	Not serious	Large effect size	168/16151 (1.0 %)	273/14888 (1.8 %)	RR 0.53 (0.41 to 0.69)	9 fewer per 1000 (from 6 fewer to 11 fewer)	Moderate	Important

### Other outcomes

None of the included studies reported data for the remaining outcomes of interest for sharps injury prevention syringes. Whitby et al. made the following statement without reporting any statistical data: “No significant increase in bloodstream infections was detected during the study period” [15].

### Meta-analyses for reuse prevention syringes

As stated earlier, three studies reported on devices that qualified as both reuse prevention devices and injury protection devices [15, 21, 22]. Therefore, we conducted subgroup analyses of those studies.

### Needlestick injuries

**Fig. 8:**
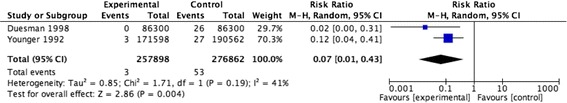
NSI per device for reuse prevention devices

NSI per device: The meta-analysis of two studies resulted in a pooled relative risk of 0.07 [0.01, 0.43] (Fig. 8) [21, 22]. The I value was 41 %.

**Fig. 9:**
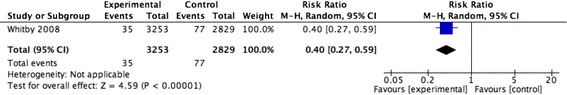
NSI per healthcare worker for reuse prevention devices

NSI per healthcare worker: Data from one eligible study indicate a RR of 0.40 [0.27, 0.59] (Fig. 9) [15]. The quality of evidence was rated as moderate (Table 2).

**Table 2:** Evidence profile for sharp reuse prevention syringes versus single use disposable syringes when used by healthcare workers to deliver intramuscular, subcutaneous or intradermal injections

Quality assessment							№ of patients		Effect		Quality	Importance
№ of studies	Study design	Risk of bias	Inconsistency	Indirectness	Imprecision	Other considerations	IM/SC/I Reuse prevention safety devices	no safety devices	Relative (95 % CI)	Absolute (95 % CI)		
HIV, HBV and HCV infections amongst patients: not reported												
												Critical
Reuse of syringes: not reported												
												Important
NSI per HCW												
1	Observational	Not serious	Not serious	Not serious	Not serious	Large effect size	35/3253 (1.1)%	77/2829 (2.7)%	RR 0.4 (0.27 to 0.59)	16 fewer per 1000 (from 11 fewer to 20 fewer)	Moderate	Important

### Other outcomes

None of the included studies reported data for the remaining outcomes of interest for reuse prevention syringes. As mentioned above, Whitby et al. made the following statement without reporting any statistical data: “No significant increase in bloodstream infections was detected during the study period” [15].

### Additional analyses

Although we planned to conduct subgroup analyses to explain heterogeneity, we did not have the opportunity to conduct them mainly because of the relatively small number of studies per analysis. Another reason is the lack of reported data on some of the factors based on which we planned to conduct the analyses: the type of device, level of expertise of HCWs, and time of injury (before, during, or after the injection).

Also, studies did not consistently report stratified outcome data by route of injection (intramuscular, intradermal, subcutaneous).

## Discussion

In summary, we identified moderate quality evidence that sharp injury prevention syringes reduce the incidence of needlestick injuries per healthcare worker. We identified no studies, meeting eligibility criteria for inclusion and reporting data for: HIV, HBV, and HCV infections amongst healthcare workers; nor studies, meeting eligibility criteria for inclsion and reporting on the effect of reuse prevention syringes on the reuse of syringes; nor HIV, HBV, and HCV infections amongst patients.

The main limitation in the literature is the lack of evaluation of the effects of the safety devices on outcomes other than needlestick injuries, whether benefits or harms.

Particularly relevant outcomes include the reuse of syringes, or blood borne infections, particularly HIV, HBV, and HCV amongst healthcare workers or patients.

Another limitation related to meta-analytical techniques, is that heterogeneity may be underestimated especially when analyzing a small number of studies [59]. That is why we opted to use the random effect model irrespective of the value of I statistic. Also, given the included studies are relatively old [60], it is likely that publication bias exists and we were underpowered to detect it. Finally, one has to consider that the observed decrease in needle stick injuries shown by the before-after studies, may reflect time trends related to factors such as changes in the legislation, hospital policies, standards of reporting of needle stick injuries.

We have identified two other systematic reviews addressing questions that are similar but not the same as our question [8, 61]. A Cochrane review addressed different types of safety devices for preventing percutaneous exposure injuries caused by needles in healthcare personnel [8]. They found “no clear evidence that the introduction of safe injection devices changed the NSI rate”. In fact, the Cochrane review included only four studies potentially relevant to our review (i.e., injection devices) [18, 25, 27, 62].

While we included three of these studies [18, 25, 27], we excluded the fourth because it was conducted in an educational setting, as opposed to a healthcare delivery setting [62]. Moreover, they analysed two of those studies separately because they reported on multiple safety devices [18, 27]. In our review, we abstracted from those two studies data specific to injection devices and included them in the meta-analysis.

In addition, we included six additional studies not identified by the Cochrane review.

Indeed, the differences in rating the quality of evidence between the Cochrane review and our review could be explained by the differences in study inclusion and the challenges with inter-rater reliability of assessing the quality of evidence [63].

Another review published by the Health and Safety Laboratory for the Health and Safety Executive 2012, addressed different types of safety devices for preventing percutaneous exposure injuries caused by needles in healthcare personnel [61]. They found “there was sufficient published evidence to consider the use of safer sharps devices to reduce the incidence of sharps injuries amongst UK healthcare workers”. In fact, this review included only seven studies potentially relevant to our review (i.e., injection devices) [10, 18, 27, 29, 39, 44, 50]. While we included three of these studies [10, 18, 27], we excluded the other four [29, 39, 44, 50] because these were evaluating intravenous injection or phlebotomy safety devices. Furthermore, they included seventeen other studies that we judged as not eligible for our review.

There is paucity of data about the cost or cost-effectiveness of introducing those devices into healthcare settings. Valls et al. reported that the introduction of sheathed needles for subcutaneous and intramuscular drug administration led to the following changes in cost: −0.010 on hospital wards per patient-day and 0.021 in the emergency department per patient [58]. Whitby et al. reported $46,000 increase in the annual budget of the hospital upon introduction of retractable syringes [15].

## Conclusions

The findings of this study have important implications for HCWs practice. Indeed, the introduction of sharps injury prevention devices into healthcare settings is likely to reduce needlestick injuries. Healthcare managers planning to introduce those devices need to consider the cost related to their introduction. They also should do that as part of a comprehensive injection safety program. Such program would include education about the risks associated with accidental injuries, training in using the safety devices, surveillance and reporting of needle stick injuries among HCWs, monitoring and evaluation of the program implementation, immunization of healthcare workers against HBV, and post exposure prophylaxis. In addition, administrators should involve healthcare workers in selecting the devices. Given the paucity of data on the effectiveness of reuse prevention syringes, healthcare managers need to consider their use mainly in settings with high rates of syringe reuse and high prevalence of blood borne pathogens.

The findings of this study have also important research implications. Future studies should assess the impact of introducing safety devices on reuse rates, and on incidence of blood borne infections amongst healthcare workers and patients. In terms of methodology, randomized trials with standardized methods for measuring incidence of sharps injuries would provide better quality evidence relative to currently available evidence. There is also a need to conduct cost-effectiveness studies for different settings, particularly low and middle income countries.

## Additional files

Additional file 1:
**Search strategies.** (PDF 15 kb) Additional file 2:
**List of excluded studies and reasons for exclusion.** (PDF 7 kb) Additional file 3:
**Characteristics of included studies.** (PDF 15 kb) Additional file 4:
**Risk of bias in the included randomized study, with each potential source of bias judged as high, low, or unclear risk.** (PDF 5 kb) Additional file 5:
**Risk of bias in the included non-randomized studies, with each potential source of bias judged as high, low, or unclear risk.** (PDF 11 kb)

## Abbreviations

CDC: Centers for Disease Control
DALY: Disability-adjusted life years
HBV: Hepatitis B virus
HCV: Hepatitis C virus
HCW: Healthcare workers
HIV: Human immunodeficiency virus
ID: Intradermal
IM: Intramuscular
NSI: Needlestick injuries
RR: Relative risk
SC: Subcutaneous
WHO: World Health Organization
