# Supplementary material for: Safety engineered injection devices for intramuscular, subcutaneous and intradermal injections in healthcare delivery settings: a systematic review and meta-analysis
Source: BMC Nurs. 2015 Dec 30;14:71. doi: 10.1186/s12912-015-0119-1 (PMC4697323; doi:10.1186/s12912-015-0119-1)
Supplement: Additional file 1: — Search strategies. (PDF 15 kb) [file 12912_2015_119_MOESM1_ESM.pdf]

Additional file 1: Search strategies.

Search strategy used in Medline:

1 Health Personnel/
2 Personnel, Hospital/
3 ((Healthcare or health-care or (health adj care)) adj2 worker*).mp.
4 Paramedic*.mp.
5 ((medical or nurs*or ancillary) adj2 staff*).mp.
6 (Medical adj2 laboratory adj2 techn*).mp.
7 Pharmacist*.mp.
8 physician*.mp.
9 Hospitalist*.mp.
10 internist*.mp.
11 doctor*.mp.
12 Phlebotomist*.mp.
13 exp Needlestick Injuries/
14 exp Accidents, Occupational/ and (syringe* or needle* or inject*).mp.
15 (injur* adj3 (syringe* or needle* or inject*)).mp.
16 exp Accidents, Occupational/ and (syringe* or needle* or inject*).mp.
17 exp Accident Prevention/ and (syringe* or needle* or inject*).mp.
18 (blood adj3 collection adj3 (syringe* or needle* or system* or device* or material* or product* or set*)).mp.
19 ((need-less or needless or needle-free or needlefree) adj3 (syringe* or needle* or system* or device* or material* or product* or set* or inject*)).mp.
20 (Single adj3 "use" adj3 (syringe* or needle* or system* or device* or material* or product* or set* or inject*)).mp.
21 (prevent* adj3 (syringe* or needle* or system* or device* or material* or product* or set* or inject*)).mp.
22 (reuse adj3 (syringe* or needle* or system* or device* or material* or product* or set* or inject*)).mp.
23 (exp Equipment Reuse/ or exp Disposable equipment/) and (syringe* or needle* or system* or device* or material* or product* or set* or inject*).mp.
24 (Disposable adj2 equipment* adj3 (syringe* or needle* or inject*)).mp.
25 ((prefill* or pre-fill*) adj3 (syringe* or needle* or inject*)).mp.
26 (Autopen or auto-pen).mp.
27 "Vetter Lyo-ject".mp.
28 Vasceze.mp.

29 Sterimatic.mp.
30 "Safe-Point".mp.
31 "Needle-Pro".mp.
32 Hypak.mp.
33 VanishPoint.mp.
34 "Slip-lock".mp.
35 Luerlok.mp.
36 "Bio-Set".mp.
37 "Auto-disposable syringe*".mp.
38 ((prefill* or pre-fill*) adj2 syringe*).mp.
39 "BD Hypak".mp.
40 "Safety-Lok".mp.
41 (Kendall's adj2 Monoject).mp.
42 "autodestruct syringe".mp.
43 SoloShot.mp.
44 "Monodose syringe*".mp.
45 "Unifine pentip*".mp.
46 Autoject.mp.
47 (ultrasafe adj passive adj delivery adj system).mp.
48 "Tip-Lok".mp.
49 "Gettig Guard".mp.
50 "Inviro SNAP!".mp.
51 "Maxxon safety syringe*".mp.
52 "monoject magellan".mp.
53 "needle-pro".mp.
54 "point-lok".mp.
55 "wandplus".mp.
56 "safetyglide".mp.
57 "safety wand".mp.
58 "powder ject".mp.
59 or/1-12
60 or/13-58
61 and/59-60

Search strategy used in EMBASE:

1 Health Personnel/
2 Personnel, Hospital/
3 ((Healthcare or health-care or (health adj care)) adj2 worker*).mp. [mp=title, abstract, subject headings, heading word, drug trade name, original title, device manufacturer, drug manufacturer, device trade name, keyword]
4 Paramedic*.mp. [mp=title, abstract, subject headings, heading word, drug trade name, original title, device manufacturer, drug manufacturer, device trade name, keyword]
5 ((medical or nurs*or ancillary) adj2 staff*).mp. [mp=title, abstract, subject headings, heading word, drug trade name, original title, device manufacturer, drug manufacturer, device trade name, keyword]
6 (Medical adj2 laboratory adj2 techn*).mp. [mp=title, abstract, subject headings, heading word, drug trade name, original title, device manufacturer, drug manufacturer, device trade name, keyword]
7 Pharmacist*.mp. [mp=title, abstract, subject headings, heading word, drug trade name, original title, device manufacturer, drug manufacturer, device trade name, keyword]
8 physician*.mp. [mp=title, abstract, subject headings, heading word, drug trade name, original title, device manufacturer, drug manufacturer, device trade name, keyword]
9 Hospitalist*.mp. [mp=title, abstract, subject headings, heading word, drug trade name, original title, device manufacturer, drug manufacturer, device trade name, keyword]
10 internist*.mp. [mp=title, abstract, subject headings, heading word, drug trade name, original title, device manufacturer, drug manufacturer, device trade name, keyword]
11 doctor*.mp. [mp=title, abstract, subject headings, heading word, drug trade name, original title, device manufacturer, drug manufacturer, device trade name, keyword]
12 Phlebotomist*.mp. [mp=title, abstract, subject headings, heading word, drug trade name, original title, device manufacturer, drug manufacturer, device trade name, keyword]
13 exp Needlestick Injuries/
14 exp Accidents, Occupational/ and (syringe* or needle* or inject*).mp. [mp=title, abstract, subject headings, heading word, drug trade name, original title, device manufacturer, drug manufacturer, device trade name, keyword]
15 (injur* adj3 (syringe* or needle* or inject*)).mp. [mp=title, abstract, subject headings, heading word, drug trade name, original title, device manufacturer, drug manufacturer, device trade name, keyword]
16 exp Accidents, Occupational/ and (syringe* or needle* or inject*).mp. [mp=title, abstract, subject headings, heading word, drug trade name, original title, device manufacturer, drug manufacturer, device trade name, keyword]
17 exp Accident Prevention/ and (syringe* or needle* or inject*).mp. [mp=title, abstract, subject headings, heading word, drug trade name, original title, device manufacturer, drug manufacturer, device trade name, keyword]
18 (blood adj3 collection adj3 (syringe* or needle* or system* or device* or material* or product* or set*)).mp. [mp=title, abstract, subject headings,

heading word, drug trade name, original title, device manufacturer, drug manufacturer, device trade name, keyword]

19 ((need-less or needless or needle-free or needlefree) adj3 (syringe* or needle* or system* or device* or material* or product* or set* or inject*)).mp. [mp=title, abstract, subject headings, heading word, drug trade name, original title, device manufacturer, drug manufacturer, device trade name, keyword]

20 (Single adj3 "use" adj3 (syringe* or needle* or system* or device* or material* or product* or set* or inject*)).mp. [mp=title, abstract, subject headings, heading word, drug trade name, original title, device manufacturer, drug manufacturer, device trade name, keyword]

21 (prevent* adj3 (syringe* or needle* or system* or device* or material* or product* or set* or inject*)).mp. [mp=title, abstract, subject headings, heading word, drug trade name, original title, device manufacturer, drug manufacturer, device trade name, keyword]

22 (reuse adj3 (syringe* or needle* or system* or device* or material* or product* or set* or inject*)).mp. [mp=title, abstract, subject headings, heading word, drug trade name, original title, device manufacturer, drug manufacturer, device trade name, keyword]

23 (exp Equipment Reuse/ or exp Disposable equipment/) and (syringe* or needle* or system* or device* or material* or product* or set* or inject*).mp. [mp=title, abstract, subject headings, heading word, drug trade name, original title, device manufacturer, drug manufacturer, device trade name, keyword]

24 (Disposable adj2 equipment* adj3 (syringe* or needle* or inject*)).mp. [mp=title, abstract, subject headings, heading word, drug trade name, original title, device manufacturer, drug manufacturer, device trade name, keyword]

25 ((prefill* or pre-fill*) adj3 (syringe* or needle* or inject*)).mp. [mp=title, abstract, subject headings, heading word, drug trade name, original title, device manufacturer, drug manufacturer, device trade name, keyword]

26 (Autopen or auto-pen).mp. [mp=title, abstract, subject headings, heading word, drug trade name, original title, device manufacturer, drug manufacturer, device trade name, keyword]

27 "Vetter Lyo-ject".mp. [mp=title, abstract, subject headings, heading word, drug trade name, original title, device manufacturer, drug manufacturer, device trade name, keyword]

28 Vasceze.mp. [mp=title, abstract, subject headings, heading word, drug trade name, original title, device manufacturer, drug manufacturer, device trade name, keyword]

29 Sterimatic.mp. [mp=title, abstract, subject headings, heading word, drug trade name, original title, device manufacturer, drug manufacturer, device trade name, keyword]

30 "Safe-Point".mp. [mp=title, abstract, subject headings, heading word, drug trade name, original title, device manufacturer, drug manufacturer, device trade name, keyword]

31 "Needle-Pro".mp. [mp=title, abstract, subject headings, heading word, drug trade name, original title, device manufacturer, drug manufacturer, device trade name, keyword]

32 Hypak.mp. [mp=title, abstract, subject headings, heading word, drug trade name, original title, device manufacturer, drug manufacturer, device trade name, keyword]

33 VanishPoint.mp. [mp=title, abstract, subject headings, heading word, drug trade name, original title, device manufacturer, drug manufacturer, device trade name, keyword]

34 "Slip-lock".mp. [mp=title, abstract, subject headings, heading word, drug trade name, original title, device manufacturer, drug manufacturer, device trade name, keyword]

35 Luerlok.mp. [mp=title, abstract, subject headings, heading word, drug trade name, original title, device manufacturer, drug manufacturer, device trade name, keyword]

36 "Bio-Set".mp. [mp=title, abstract, subject headings, heading word, drug trade name, original title, device manufacturer, drug manufacturer, device trade name, keyword]

37 "Auto-disposable syringe*".mp. [mp=title, abstract, subject headings, heading word, drug trade name, original title, device manufacturer, drug manufacturer, device trade name, keyword]

38 ((prefill* or pre-fill*) adj2 syringe*).mp. [mp=title, abstract, subject headings, heading word, drug trade name, original title, device manufacturer, drug manufacturer, device trade name, keyword]

39 "BD Hypak".mp. [mp=title, abstract, subject headings, heading word, drug trade name, original title, device manufacturer, drug manufacturer, device trade name, keyword]

40 "Safety-Lok".mp. [mp=title, abstract, subject headings, heading word, drug trade name, original title, device manufacturer, drug manufacturer, device trade name, keyword]

41 (Kendall's adj2 Monoject).mp. [mp=title, abstract, subject headings, heading word, drug trade name, original title, device manufacturer, drug manufacturer, device trade name, keyword]

42 "autodestruct syringe".mp. [mp=title, abstract, subject headings, heading word, drug trade name, original title, device manufacturer, drug manufacturer, device trade name, keyword]

43 SoloShot.mp. [mp=title, abstract, subject headings, heading word, drug trade name, original title, device manufacturer, drug manufacturer, device trade name, keyword]

44 "Monodose syringe*".mp. [mp=title, abstract, subject headings, heading word, drug trade name, original title, device manufacturer, drug manufacturer, device trade name, keyword]

45 "Unifine pentip*".mp. [mp=title, abstract, subject headings, heading word, drug trade name, original title, device manufacturer, drug manufacturer, device trade name, keyword]

46 Autoject.mp. [mp=title, abstract, subject headings, heading word, drug trade name, original title, device manufacturer, drug manufacturer, device trade name, keyword]

47 (ultrasafe adj passive adj delivery adj system).mp. [mp=title, abstract, subject headings, heading word, drug trade name, original title, device manufacturer, drug manufacturer, device trade name, keyword]

48 "Tip-Lok".mp. [mp=title, abstract, subject headings, heading word, drug trade name, original title, device manufacturer, drug manufacturer, device trade name, keyword]

49 "Gettig Guard".mp. [mp=title, abstract, subject headings, heading word, drug trade name, original title, device manufacturer, drug manufacturer, device trade name, keyword]
50 "Inviro SNAP!".mp. [mp=title, abstract, subject headings, heading word, drug trade name, original title, device manufacturer, drug manufacturer, device trade name, keyword]
51 "Maxxon safety syringe*".mp. [mp=title, abstract, subject headings, heading word, drug trade name, original title, device manufacturer, drug manufacturer, device trade name, keyword]
52 "monoject magellan".mp. [mp=title, abstract, subject headings, heading word, drug trade name, original title, device manufacturer, drug manufacturer, device trade name, keyword]
53 "needle-pro".mp. [mp=title, abstract, subject headings, heading word, drug trade name, original title, device manufacturer, drug manufacturer, device trade name, keyword]
54 "point-lok".mp. [mp=title, abstract, subject headings, heading word, drug trade name, original title, device manufacturer, drug manufacturer, device trade name, keyword]
55 "wandplus".mp. [mp=title, abstract, subject headings, heading word, drug trade name, original title, device manufacturer, drug manufacturer, device trade name, keyword]
56 "safetyglide".mp. [mp=title, abstract, subject headings, heading word, drug trade name, original title, device manufacturer, drug manufacturer, device trade name, keyword]
57 "safety wand".mp. [mp=title, abstract, subject headings, heading word, drug trade name, original title, device manufacturer, drug manufacturer, device trade name, keyword]
58 "powder ject".mp. [mp=title, abstract, subject headings, heading word, drug trade name, original title, device manufacturer, drug manufacturer, device trade name, keyword]
59 or/1-12
60 or/13-58
61 and/59-60

Search strategy used in CENTRAL:

1 MeSH descriptor: [Health Personnel] explode all trees
2 MeSH descriptor: [Personnel, Hospital] explode all trees
3 ((Healthcare or health-care or (health near/1 care)) near/2 worker*):ti,ab,kw (Word variations have been searched)
4 Paramedic*:ti,ab,kw (Word variations have been searched)
5 ((medical or nurs*OR ancillary) near/2 staff*):ti,ab,kw (Word variations have been searched)
6 (Medical near/2 laboratory near/2 techn*):ti,ab,kw (Word variations have been searched)
7 Pharmacist*:ti,ab,kw (Word variations have been searched)
8 physician*:ti,ab,kw (Word variations have been searched)
9 Hospitalist*:ti,ab,kw (Word variations have been searched)
10 internist*:ti,ab,kw (Word variations have been searched)
11 doctor*:ti,ab,kw (Word variations have been searched)
12 Phlebotomist*:ti,ab,kw (Word variations have been searched)
13 MeSH descriptor: [Needlestick Injuries] explode all trees
14 MeSH descriptor: [Accidents, Occupational] explode all trees
15 (syringe* or needle* or inject*):ti,ab,kw (Word variations have been searched)
16 #14 and #15
17 (injur* near/3 (syringe* or needle* or inject*)):ti,ab,kw (Word variations have been searched)
18 MeSH descriptor: [Accident Prevention] explode all trees
19 (syringe* or needle* or inject*):ti,ab,kw (Word variations have been searched)
20 #18 and #19
21 (blood near/3 collection near/3 (syringe* or needle* or system* or device* or material* or product* or set*)):ti,ab,kw (Word variations have been searched)
22 ((need-less or needless or needle-free or needlefree) near/3 (syringe* or needle* or system* or device* or material* or product* or set* or inject*)):ti,ab,kw (Word variations have been searched)
23 (Single near/3 "use" near/3 (syringe* or needle* or system* or device* or material* or product* or set* or inject*)):ti,ab,kw (Word variations have been searched)
24 (prevent* near/3 (syringe* or needle* or system* or device* or material* or product* or set* or inject*)):ti,ab,kw (Word variations have been searched)
25 (reuse near/3 (syringe* or needle* or system* or device* or material* or product* or set* or inject*)):ti,ab,kw (Word variations have been searched)
26 MeSH descriptor: [Equipment Reuse] explode all trees
27 MeSH descriptor: [Disposable Equipment] explode all trees
28 #26 or #27
29 (syringe* or needle* or system* or device* or material* or product* or set* or inject*):ti,ab,kw (Word variations have been searched)
30 #28 and #29
31 (Disposable near/2 equipment* near/3 (syringe* or needle* or inject*)):ti,ab,kw (Word variations have been searched)
32 ((prefill* or pre-fill*) near/3 (syringe* or needle* or inject*)):ti,ab,kw (Word variations have been searched)

33 ((prefill* or pre-fill*) near/3 (syringe* or needle* or inject*)):ti,ab,kw (Word variations have been searched)
34 (Autopen or auto-pen):ti,ab,kw (Word variations have been searched)
35 "Vetter Lyo-ject":ti,ab,kw (Word variations have been searched)
36 Vasceze:ti,ab,kw (Word variations have been searched)
37 Sterimatic:ti,ab,kw (Word variations have been searched)
38 "Safe-Point":ti,ab,kw (Word variations have been searched)
39 "Needle-Pro":ti,ab,kw (Word variations have been searched)
40 Hypak:ti,ab,kw (Word variations have been searched)
41 VanishPoint:ti,ab,kw (Word variations have been searched)
42 Slip-lock:ti,ab,kw (Word variations have been searched)
43 Luerlok:ti,ab,kw (Word variations have been searched)
44 "Bio-Set":ti,ab,kw (Word variations have been searched)
45 "Auto-disposable syringe*":ti,ab,kw (Word variations have been searched)

46 ((prefill* or pre-fill*) near/3 syringe*):ti,ab,kw (Word variations have been searched)
47 "BD Hypak":ti,ab,kw (Word variations have been searched)
48 "Safety-Lok":ti,ab,kw (Word variations have been searched)
49 (Kendall's adj2 Monoject):ti,ab,kw (Word variations have been searched)
50 "autodestruct syringe":ti,ab,kw (Word variations have been searched)
51 SoloShot:ti,ab,kw (Word variations have been searched)
52 "Monodose syringe*":ti,ab,kw (Word variations have been searched)
53 "Unifine pentip*":ti,ab,kw (Word variations have been searched)
54 Autoject:ti,ab,kw (Word variations have been searched)
55 (ultrasafe near passive near delivery near system):ti,ab,kw (Word variations have been searched)
56 "Tip-Lok":ti,ab,kw (Word variations have been searched)
57 "Gettig Guard":ti,ab,kw (Word variations have been searched)
58 "Inviro SNAP!":ti,ab,kw (Word variations have been searched)
59 "Maxxon safety syringe*":ti,ab,kw (Word variations have been searched)
60 "monoject magellan":ti,ab,kw (Word variations have been searched)
61 "needle-pro":ti,ab,kw (Word variations have been searched)
62 "point-lok":ti,ab,kw (Word variations have been searched)
63 "wandplus":ti,ab,kw (Word variations have been searched)
64 "safetyglide":ti,ab,kw (Word variations have been searched)
65 "safety wand":ti,ab,kw (Word variations have been searched)
66 "powder ject":ti,ab,kw (Word variations have been searched)
67 #1 or #2 or #3 or #4 or #5 or #6 or #7 or #8 or #9 or #10 or #11 or #12
68 #13 or #16 or #17 or #20 or #21 or #22 or #23 or #24 or #25 or #30 or #31 or #32 or #33 or #34 or #35 or #36 or #37 or #38 or #39 or #40 or #41 or #42 or #43 or #44 or #45 or #46 or #47 or #48 or #49 or #50 or #51 or #52 or #53 or #54 or #55 or #56 or #57 or #58 or #59 or #60 or #61 or #62 or #63 or #64 or #65 or #66
69 #67 and #68

Search strategy used in CINAHL:

1 (MH "Health Personnel+")
2 (Healthcare or health-care or (health W care)) N2 worker*
3 Paramedic*
4 (medical or nurs*or ancillary) N2 staff*
5 (Medical N2 laboratory N2 techn*)
6 Pharmacist*
7 physician*
8 Hospitalist*
9 internist*
10 doctor*
11 Phlebotomist*
12 (MH "Needlestick Injuries")
13 (MH "Occupational-Related Injuries")
14 (MH "Accidents, Occupational")
15 injur* N3 (syringe* or needle* or inject*)
16 (blood N3 collection) N3 (syringe* or needle* or system* or device* or material* or product* or set*)
17 (need-less or needless or needle-free or needlefree) N3 (syringe* or needle* or system* or device* or material* or product* or set* or inject*)
18 Single N3 "use" N3 (syringe* or needle* or system* or device* or material* or product* or set* or inject*)
19 prevent* N3 (syringe* or needle* or system* or device* or material* or product* or set* or inject*)
20 (MH "Equipment Reuse")
21 (MH "Disposable Equipment")
22 (MH "Sharps Disposal")
23 (MH "Equipment Safety")
24 (syringe* or needle* or system* or device* or material* or product* or set* or inject*)
25 S20 OR S21 OR S22 OR S23
26 S24 AND S25
27 Disposable N2 equipment* N3 (syringe* or needle* or inject*)
28 (Disposable N2 equipment*) N3 (syringe* or needle* or inject*)
29 Sharp*
30 (prefill* or pre-fill*) N3 (syringe* or needle* or inject*)
31 Autopen or auto-pen
32 "Vetter Lyo-ject"
33 Vasceze
34 Sterimatic
35 "Safe-Point"
36 "Needle-Pro"
37 Hypak
38 "Slip-lock"
39 Luerlok
40 "Bio-Set"
41 "Auto-disposable syringe*
42 (prefill* or pre-fill*) N2 syringe*
43 "Safety-Lok"

44	"BD Hypak"
45	Kendall's N2 Monoject
46	"autodestruct syringe"
47	autodestruct w syringe
48	SoloShot
49	"Monodose syringe*"
50	Monodose W syringe*
51	"Unifine pentip*"
52	Autoject
53	ultrasafe N passive N delivery N system
54	"Tip-Lok"
55	"Gettig Guard"
56	"Inviro SNAP!"
57	"Maxxon safety syringe*"
58	"monoject magellan"
59	"needle-pro"
60	"point-lok"
61	"wandplus"
62	"safetyglide"
63	"powder ject"
64	S1 OR S2 OR S3 OR S4 OR S5 OR S6 OR S7 OR S8 OR S9 OR S10 OR S11
65	S12 OR S13 OR S14 OR S15 OR S16 OR S17 OR S18 OR S19 OR S26 OR S27 OR S28 OR S29 OR S30
66	S31 OR S32 OR S33 OR S34 OR S35 OR S36 OR S37 OR S38 OR S39 OR S40 OR S42 OR S43 OR S44 OR S45 OR S46 OR S47 OR S48 OR S50 OR S51 OR S52 OR S53 OR S54 OR S55 OR S58 OR S59 OR S60 OR S61 OR S62 OR S63 OR S65
67	S65 OR S66
68	S64 AND S67
