# Supplementary material for: Safety engineered injection devices for intramuscular, subcutaneous and intradermal injections in healthcare delivery settings: a systematic review and meta-analysis
Source: BMC Nurs. 2015 Dec 30;14:71. doi: 10.1186/s12912-015-0119-1 (PMC4697323; doi:10.1186/s12912-015-0119-1)
Supplement: Additional file 2: — List of excluded studies and reasons for exclusion. (PDF 7 kb) [file 12912_2015_119_MOESM2_ESM.pdf]

Additional file 2: List of excluded studies and reasons for exclusion.

**Study name**	**Reason for exclusion**
Adams 2003 [23]	Reporting on preferences, acceptability or feasibility
Azar-Cavanagh 2007[24]	Evaluating intravenous injection or phlebotomy safety devices
Beason 1992[25]	Evaluating intravenous injection or phlebotomy safety devices
Billiet 1991[28]	Evaluating intravenous injection or phlebotomy safety devices
Dugger 1992[30]	Reporting economic analysis
Edwards 2012[31]	Evaluating intravenous injection or phlebotomy safety devices
Ford 2011[55]	Reporting on preferences, acceptability or feasibility
Gartner 1992[32]	Evaluating intravenous injection or phlebotomy safety devices
Gershon 1999[33]	Evaluating intravenous injection or phlebotomy safety devices
Gil 2006 [27]	Reporting economic analysis
Gomez 2010 [29]	Evaluating intravenous injection or phlebotomy safety devices
LaMontagne 2007[34]	Evaluating intravenous injection or phlebotomy safety devices; also “quantitative use of SEDs could not be precisely estimated for intramuscular or subcutaneous injections”
Laufer 1994 [35]	Reporting economic analysis
Lawrence 1997 [36]	Evaluating intravenous injection or phlebotomy safety devices
L’Ecuyer 1996 [37]	Evaluating intravenous injection or phlebotomy safety devices
MacPherson 1996[38]	Evaluating intravenous injection or phlebotomy safety devices
McCleary 2002[39]	Evaluating intravenous injection or phlebotomy safety devices
Mendelson 1998[40]	Evaluating intravenous injection or phlebotomy safety devices
MMWR 1997[26]	Evaluating intravenous injection or phlebotomy safety devices
Mulherin 1996 [41]	Reporting on preferences, acceptability or feasibility
Orenstein 1995 [42]	Reporting on preferences, acceptability or feasibility
Peate 2001[52]	Evaluating glucometer lancets
Pereira 2010[43]	Reporting economic analysis
Puro 2010 [44]	Evaluating intravenous injection or phlebotomy safety devices
Rogues 2004 [45]	Evaluating intravenous injection or phlebotomy safety devices
Skolnick 1993 [46]	Evaluating intravenous injection or phlebotomy safety devices
Suzuki 2006 [56]	Evaluating intravenous injection or phlebotomy safety devices
Terrell 1993 [47]	Evaluating intravenous injection or phlebotomy safety devices
Vaudelle-Malbos 1996[48]	Reporting on preferences, acceptability or feasibility
Wolfrum 1994 [49]	Evaluating intravenous injection or phlebotomy safety devices
Yassi 1995 [50]	Evaluating intravenous injection or phlebotomy safety devices
Zakrzewska[51]	Reporting data on SED not in the HCW setting
