# Supplementary material for: Safety engineered injection devices for intramuscular, subcutaneous and intradermal injections in healthcare delivery settings: a systematic review and meta-analysis
Source: BMC Nurs. 2015 Dec 30;14:71. doi: 10.1186/s12912-015-0119-1 (PMC4697323; doi:10.1186/s12912-015-0119-1)
Supplement: Additional file 3: — Characteristics of included studies. (PDF 15 kb) [file 12912_2015_119_MOESM3_ESM.pdf]

Additional file 3: Characteristics of included studies.

**Study Name and focus**	**Device**	**Funding and conflicts of interest**	**Study Design**	**Participants, setting**	**Intervention**	**Control**	**Outcomes**
Younger 1992 [15]<br><br>Focus: Injury prevention and reuse prevention	Monoject™ Safety Syringe (Sherwood Medical). It consists of single use, 3 cc shielded syringe with hollow bore needle and a retractable plastic shield and restrictive collar.	Funding: "study supported in part by Sherwood Medical, St. Louis, Missouri". Conflicts of interest: not reported.	Surveillance study, most likely prospective. Pre and post intervention analysis of NSI rates (Pre: April - July 1990; Post: July - October 1990).	9001 healthcare workers at three US medical centers (University of Virginia Health Sciences Center, a major public hospital and Pacific Presbyterian Medical Center).	Introduction of Monoject™ Safety Syringe. Workers received training (for ~ 2 weeks) in proper use and disposal of the 3 cc shielded syringe.	"Standard 3 cc syringe".	Incidence of NSI (self-reported) among HCWs.
Duesman 1998 [17]<br><br>Focus: Injury prevention	VanishPoint®, a 3cc automated retraction syringe: "this causes the syringe's patented spring	Funding: not clearly reported; but "syringes had been purchased by the facilities". Conflicts of	Retrospective study (survey collected data between Jan 9 and 13, 1998). Pre-Post Intervention NSI rates. Pre:	All HCWs of 26 medical facilities: 10 acute care, 7 psychiatric-drug rehabilitation, 3 Public	Implementation of Vanishpoint®, in all surveyed facilities accompanied with training given during the	Commercially available 3cc syringe with a standard non-removable	Incidence of NSI (self-reporting to an employee assigned to document all reported NSIs) among HCWs.

and reuse prevention	mechanism to retract the needle directly from the patient into the empty syringe barrel after medication is administered.”	interest: not reported.	1996-1997; Post: 1997-1998.	Health, 2 correctional, 2 family/primary care, 1 research, and occupational health facilities. Further details about medical facilities not provided; most likely United States.	product evaluation period (throughout 1997).	needle.	
Reddy 2001 [13]<br><br>Focus: Injury prevention<br><br>*Also assessed intravenous systems; data not provided separately for different devices	“Safety syringes and needless intravenous systems” (no further details provided).	Funding: not reported. Conflicts of interest: not reported.	Retrospective study design. Before and after collection of data on NSI; Pre: 1994 – 1997; Post: 1997 – 1999.	HCW in nearly all departments in an 800 plus bed hospital in Texas, United States, including HCW with direct patient contact and ancillary workers; physicians not included.	Implementation of safety engineered devices.	“Traditional needled devices.”	Rates of NSI.

Sohn 2004 [19]<br><br>Focus: Injury prevention<br><br>*Also assessed intravenous systems; data provided separately for different devices	"Safer-needle system" was composed of "variety of safety-engineered devices from seven manufacturers to allow for needle-safe intravenous (IV) delivery, blood collection, IV insertion, and intramuscular and subcutaneous injection".	Funding: National Institute of Allergy and Infectious Diseases; the Centers for Disease Control and Prevention; and the Prevention Epicenters Program. Conflicts of interest: not reported.	Prospective study. Before-and-after intervention trial: before: Jan 1998–Dec 2000; after: Feb 2001– Jan 2002.	Staff (about 4000 FTEs per year) at a 427-bed, tertiary-care hospital in Manhattan, United States.	Implementation of the "safer-needle system". All HCWs responsible for direct patient care were required to attend a training session in the months preceding implementation.	Unspecified "conventional devices".	Incidence of percutaneous injuries (self-reported).
Adams 2006 [14]<br><br>Focus: Injury prevention	Safety needle devices consisted of hypodermic needle devices with safety feature requiring activation: Safety Glide™	Funding: educational grant by Becton Dickinson, Oxford, UK. Conflicts of interest: not reported.	Prospective cohort study. A four-year (2001-2004), pre and post intervention analysis of NSI rate.	All HCW from 4 clinical areas (2 surgical, 1 medical and 1 outpatient) at the University Hospital Birmingham NHS	2002: enhanced training program. 2003:pilot evaluation of the safety needle devices. Late 2003 throughout 2004:	"Standard hypodermic needles" (2001); although standard needles used in 2002 and 2003 there	Incidence of NSI (data collected prospectively 2001 onwards from reports to the Trust's occupational health and safety

*Also assessed needles and blunt fill cannulae that appear to be for phlebotomy use; data not provided separately for different devices	needles, SafetyGlide™ TNT insulin units, blunt fill cannulae (Becton Dickinson, Temse, Belgium).			Foundation Trust (number of participants not reported), United Kingdom.	Introduction of the safety needle devices.	were co-interventions during that year (enhanced training and pilot evaluation)	department and risk management.
Valls 2007 [22]<br><br>Focus: Injury prevention<br><br>*Also assessed	Safety-engineered devices. Needles with sheaths for intramuscular and subcutaneous administration of drugs (Eclipse™, Becton-Dickinson;	Funding: Directorate General of Public Health of the Autonomous Community of Valencia, Spain. Conflicts of interest: "all authors report no conflicts	Prospective "quasi experimental" trial. Before-and-after intervention evaluation. Pre: October 2004 - March 2005; Post: October 2005 - March	75 nurses working on selected (and not all) wards of Hospital Virgen de la Salud-Elda; Alicante, Spain.	Safety-engineered devices introduced in October 2005. Nurses participated in a 2-hour training experience about the devices to be tested and ways to improve	Unspecified "conventional devices".	Incidence of NSI (voluntary self-reporting complimented with active surveillance and reporting by nurses in charge) among HCWs.

vacuum phlebotomy systems, blood-gas syringes, lancets (with retractable, single-use puncture sticks), and intravascular catheters; data provided separately for different devices	Surshield™, Terumo).	of interest relevant to this article".	2006.		compliance in both the use of the devices and the reporting of injuries. An additional 15 minutes of on-site training was carried out each time a new device was tested.		
Whitby 2008 [16] Focus: Injury prevention and reuse	Safety engineered devices, including retractable syringes (VanishPoint®, a subcutaneous device).	Funding: not reported Conflicts of interest: not clearly reported.	Prospective study design. Before and after collection of data on NSI; Pre: 2000-2004; post: 2005-2006.	Full-time employee and part-time HCW from multiple occupational groups within the hospital including medical,	Introduction of the safety engineered devices.	Unspecified needle devices.	Risk of needle stick injury.

prevention<br><br>*Also assessed needle-free intravenous systems, and safety winged butterfly needles; data provided separately for different devices				nursing, hotel services, and ther in an 800-bed university teaching. Princess Alexandra Hospital, Brisbane, Australia.			
Van der Molen 2011[20]<br><br>Focus: Injury prevention	Eclipse™ by Becton Dickinson. According to manufacturer website, it has a “pivoting needle technology” and a safety cover. It can be used for	Funding: Dutch Ministry of Social Affairs and Employment support. Conflicts of interest: not reported.	Three-armed cluster prospective randomized controlled trial. T0: baseline in December 2006, T1: 6 months follow-up and T2: 12	495 workers from 23 wards from 4 hospital divisions in the Academic Medical Center, University of Amsterdam,	Introduction of an injection needle with the safety device with a needle safety workshop (one-hour interactive PowerPoint presentation).	Needle safety workshop without the introductio n of the new device (previous device unspecified); 3rd arm (control)	Incidence of NSI (self-reported NSI over the previous 6 months). We did not use data from hospital register as this was not the primary outcome in the

	both injection and blood collection.		months follow-up.	Netherlands (out of 796 potential participants; 62%response rate).		did not include safety devices or workshop.	study.
Hoffman 2013 [21]<br><br>Focus: Injury prevention	Hypodermic needles and lancets. The safety mechanism was triggered actively for the hypodermic needles while passively for the lancets.	Funding: not reported. Conflicts of interest: “authors declared that they have no Conflicts of interest”.	Most likely retrospective study.<br>Pre and post intervention analysis of NSI rate (1 year before and 2 year after; 2007-2009).	6493 and 6683 full-time HCWs in 2007 and 2009 respectively. Included nurses, doctors, lab technicians and students at the University Hospital Heidelberg in Germany.	Implementation of the safety devices throughout the hospital with simultaneous mandatory training for all healthcare personnel in 2008.	Unspecified needle devices.	Incidence of NSI (self-reported) amongst HCW.
