# Supplementary material for: Safety engineered injection devices for intramuscular, subcutaneous and intradermal injections in healthcare delivery settings: a systematic review and meta-analysis
Source: BMC Nurs. 2015 Dec 30;14:71. doi: 10.1186/s12912-015-0119-1 (PMC4697323; doi:10.1186/s12912-015-0119-1)
Supplement: Additional file 4: — Risk of bias in the included randomized study, with each potential source of bias judged as high, low, or unclear risk. (PDF 5 kb) [file 12912_2015_119_MOESM4_ESM.pdf]

Additional file 4: Risk of bias in the included randomized study, with each potential source of bias judged as high, low, or unclear risk.

**Study**	**Random sequence generation**	**Allocation concealment**	**Blinding**	**Completeness of data**	**Intention to treat analysis**	**Selective outcome reporting**
Van der Molen 2011 [20]	- Low risk<br><br>“The stratified allocation … was performed using a random, computerized allocation procedure.”	-Low risk<br><br>“The primary researcher (HM) blindly randomized the groups for the hospital wards and workshop teachers as well as the needle supplier and the needle-use trainers”	-High risk<br><br>“The intervention and implementation strategy made blind group assignment impossible for the participating wards, the workers and the trainers”	-High risk<br><br>“The largest limitation of this study was the high proportion of workers lost to follow-up. At the cluster level, all wards remained in the study, but at the individual level 49% were lost at 12 months follow-up for the main outcome measure of self-reported NSIs compared to baseline”	-Low risk<br><br>“The analysis were performed on an intention-to-treat basis”	-Low risk<br><br>Outcomes listed in trial registry (NTR1207) and in the methods section are reported in the results section
