# Supplementary material for: Safety engineered injection devices for intramuscular, subcutaneous and intradermal injections in healthcare delivery settings: a systematic review and meta-analysis
Source: BMC Nurs. 2015 Dec 30;14:71. doi: 10.1186/s12912-015-0119-1 (PMC4697323; doi:10.1186/s12912-015-0119-1)
Supplement: Additional file 5: — Risk of bias in the included non-randomized studies, with each potential source of bias judged as high, low, or unclear risk. (PDF 11 kb) [file 12912_2015_119_MOESM5_ESM.pdf]

Additional file 5: Risk of bias in the included non-randomized studies, with each potential source of bias judged as high, low, or unclear risk.

Study Name	Developing and applying appropriate eligibility criteria	Measurement of intervention	Measurement of outcome	Controlling for confounding	Completeness of data
Younger 1992 [15]	-Low risk<br>Participants in the pre and post periods were selected from the same population.	- Low risk<br><br>"All standard 3 cc syringes were removed from service during the study phase to assure universal use of the safety syringe at the three centers".<br><br>"Workers on all three shifts received training and New workers employed after this time received individual instruction in the use of the safety syringe"	- Low risk<br><br>Employees report all NSI to their supervisors immediately; after that the institution's employee health service evaluate the employee. Follow up of the employees occurred at the health service; and records of NSIs are maintained.	- Unclear risk<br><br>No information on how confounding was handled.	- Unclear Risk.<br><br>Authors did not report any missing data

Duesman 1998 [17]	- Low Risk<br><br>Participants of the pre-post periods belong to the same population.	- High Risk<br><br>“Each of the facilities surveyed had used the test syringe for at least three months and had purchased a minimum of 500 syringes for use during the study period- the 12 months of calendar year 1997.”	- Unclear Risk<br><br>Data collected by phone from the facility’s “employee responsible for documenting reported accidental needles ticks or an employee privy to that information”	- Unclear Risk<br><br>No information on how confounding was handled.	- Unclear Risk<br><br>No missing Data mentioned.
Reddy 2001 [13]	-Low risk<br>Participants selected from the same hospital in the pre and post periods<br><br>Participation rate: not mentioned	- High risk<br><br>“During the course of the post implementation period, traditional needle syringes and traditional intravenous catheter systems still existed.”	-Unclear risk<br>“Retrospective data were composed of injury reports from employees who reported an occupational injury to the hospital’s Occupational Health Clinic”	-Unclear risk<br>No information on how confounding was handled.<br>One potential confounder is an extensive in-service in 1195 (in the middle of the pre phase) “to educate all hospital employees on the importance of needle stick safety and blood borne pathogens"	-Unclear risk<br>Authors did not report any missing data

Sohn 2004 [19]	-Low risk<br>Participants from the same hospital in the pre and post period	- Low risk<br><br>Prior to the program's implementation, less than 2% of devices in use were safety devices	-Low risk<br>Prospectively collected data on needle stick injuries	-Unclear risk<br>No information on how confounding was handled.	-Unclear risk<br>Authors did not report any missing data
Adams 2006 [14]	-Low risk.<br><br>Participants selected from the same population in the pre and post period	- Low risk<br><br>"Standard needles were removed except for the cardiac arrest trolley, as members of the cardiac arrest team may not all have been familiar with the safety devices"<br><br>" On completion of the training programme, the safety needles were introduced into the clinical areas"	-Low risk<br><br>"Prospective NSI data were captured from 2001 onwards from reports to the Trust's occupational health and safety department and risk management"	-Unclear risk<br><br>No information on how confounding was handled.<br><br>We (systematic review team) attempted to minimize confounding by focusing on the data from 2001 and 2004	- Unclear risk<br><br>Authors did not report any missing data

Valls 2007 [22]	-Low risk<br>Participants of the Pre-Post periods belong to the same population<br><br>“We selected fall and winter months, to assure continuity among the employees involved in the study”	-Low risk<br><br>“Insulin injection systems (“insulin pens”) were<br><br>the only nonsafety needle devices that HCWs were allowed to use”.,	-Low risk<br><br>“The nurses in charge of the study carried out active surveillance and reporting of injuries during the intervention period.”<br><br>Also, educational activities focused on the importance of reporting injuries.	- Unclear risk<br><br>Not clear that all confounding variables were controlled for.<br><br>“We tried to maintain control of possible prognostic variables involved in the study by selecting the same period of the year and by conducting the study in hospital areas with homogeneously distributed activities”	-Unclear risk<br><br>Authors did not report any missing data
Whitby 2008[16]	-Low risk<br>HCW from the same hospital included in the before and after phases of the study<br><br>Participation rate: not mentioned	-Low risk<br>At the commencement of the intervention in 2005, all conventional syringes and needles that were no longer required were physically removed.	-Low risk<br>The same system of reporting of NSI to the Infectious Diseases Department, which has been in place since 1996, was used throughout the study.<br><br>All data pertaining to this study were collected prospectively	-Unclear risk<br>No information on how confounding was handled.	- Unclear risk<br>Authors did not report any missing data

Hoffman 2013 [21]	-Low risk<br><br>Participants of the pre-post periods belong to the same population although the population size increased slightly with time	- Low risk<br><br>“Safety devices were introduced throughout the hospital including all departments and all operating rooms”; “training was performed in all departments and was obligatory for all healthcare personnel when the new device was introduced”	- Low risk<br><br>“Data was extracted from mandatory needlestick report”.	-Unclear risk<br><br>No information on how confounding was handled.<br><br>“The study did not cover degree of extended work shifts, time pressure or under-staffing influencing the number of needlestick infections”	- Unclear risk<br><br>Authors did not report any missing data
